# Draft Genome Sequence of *Penicillium expansum* Strain R19, Which Causes Postharvest Decay of Apple Fruit

**DOI:** 10.1128/genomeA.00635-14

**Published:** 2014-06-19

**Authors:** Jiujiang Yu, Wayne M. Jurick, Huansheng Cao, Yanbin Yin, Verneta L. Gaskins, Liliana Losada, Nikhat Zafar, Maria Kim, Joan W. Bennett, William C. Nierman

**Affiliations:** a Department of Agriculture, ARS, Beltsville Agricultural Research Center, Beltsville, Maryland, USA; b Department of Biological Sciences, Northern Illinois University, DeKalb, Illinois, USA; c The J. Craig Venter Institute, Rockville, Maryland, USA; d Department of Plant Biology and Pathology, Rutgers University, New Brunswick, New Jersey, USA

## Abstract

Among the species that cause blue mold, isolates of *Penicillium expansum* are the most prevalent and virulent species, causing more than 50 percent of postharvest decay. We report the draft genome sequence of *P. expansum* R19 in order to identify fungal virulence factors and to understand the mechanism of infection.

## GENOME ANNOUNCEMENT

*Penicillium* spp. cause postharvest decay of some fruit and have a worldwide distribution (1). *P. expansum* is the most virulent and economically significant pathogen within the genus because it causes blue mold in apples during storage and produces many different mycotoxins that impact human health (1). Postharvest losses in the United States were estimated at $4.4 million in 1992. To understand the genetic mechanism contributing to fungal virulence, spore germination, and mycotoxin production, the genome of the wild-type strain of *P. expansum* (R19) was sequenced and annotated.

Spores of *P. expansum* (R19) isolated in 2011 from a decomposing red delicious apple in Carlisle, PA, were inoculated in potato dextrose broth (PDB) at 25°C for 7 days. Genomic DNA was prepared with a DNeasy Plant Maxi Kit (Qiagen) according to the manufacturer’s instructions. Paired-end 250-bp Illumina fragment reads were generated using an Illumina MiSeq Benchtop Sequencer. The sequence depth reached 27. All reads were used to generate assemblies with Velvet Optimizer, version 2.2.0. The resulting assembly had 1,231 contigs, with an *N*_50_ value of 48,518 bp. Based on our data, the calculated genome size of *P. expansum* (R19) contained 31,415,732 bp. The G+C content of the genome was 48.24%. This is consistent with the genome size reported previously for *P. chrysogenum* (2).

The genome sequence of *P. expansum* (R19) was annotated using the MAKER program (3), which masks repeat regions with RepeatMasker as well as RepeatRunner. Putative genes were predicted by AUGUSTUS (4) trained with *Aspergillus oryzae* sequences. The predicted genes were then annotated using Interproscan 5, and *P. chrysogenum* Wisconsin 54-1255 was used as a reference for the final annotation (2).

Preliminary annotation results demonstrated that the *P. expansum* R19 genome harbors 10,554 predicted genes, with an average gene length of 1,599 bp. The total length of the coding sequence (genes) is 16,873,185 bp, which makes up 53.70% of the genome. There are 120 tRNA genes and 48 5S rRNA genes, respectively, as predicted by tRNAscan-SE 1.21 (5) and RNAmmer (6). The gene ontology (GO) analysis indicated that a total of 6,831 proteins fall in 985 major GO terms. It is estimated that there are 59 gene clusters putatively involved in the biosynthesis of secondary metabolites, as predicted by SMURF (7). This is similar to the result predicted by the AntiSMASH program (8), which resulted in 57 clusters. It is expected to identify groups of genes that are putatively involved in spore germination and fungal mycelial growth, as well as genes involved in mycotoxin biosynthesis.

### Nucleotide sequence accession numbers.

The genome sequence of *P. expansum* R19 has been deposited at DDBJ/EMBL/GenBank under the accession JHUC00000000. The version described in this paper is version JHUC01000000.
